# Fact-Checking Large Language Model Responses to a Health Care Prompt: Comparative Study

**DOI:** 10.2196/68223

**Published:** 2026-04-15

**Authors:** Padhraig Ryan, Orla Davoren, Glyn Elwyn

**Affiliations:** 1 Pharmaceutical Society of Ireland, Dublin, Ireland; 2 Dartmouth Institute for Health Policy and Clinical Practice, Dartmouth College, 1 Medical Center Drive, Williamson Translational Research Building, Level 5, Lebanon, NH, 03756, United States, 1 603-646-5678

**Keywords:** machine learning, artificial intelligence, AI, deep learning, patient medication knowledge, patient participation, shared decision-making

## Abstract

**Background:**

Large language models use machine learning to produce natural language. These models have a range of potential applications in health care, such as patient education and diagnosis. However, evaluations of large language models in health care are still scarce.

**Objective:**

This study aimed to (1) evaluate the accuracy and efficiency of automated fact-checking by 2 large language models and (2) illustrate a process through which a large language model might support a patient in redrafting a prompt to include key information needed for patient safety.

**Methods:**

A parallel comparison of 2 large language models and 3 human experts was conducted. A clinical scenario was devised in which a woman aged 23 years questions the safety of retinoid treatment for acne by sending prompts to 2 large language models (GPT-4o and OpenBioLLM-70B). GPT-4o and OpenBioLLM-70B were asked to suggest improvements to the patient’s initial prompt to elicit key information for clinical decision-making. After the patient sent the revised prompt to the large language models, the models were then asked to fact-check the final response. To test the generalizability of automated fact-checking, a set of 20 clinical statements on disparate topics, mostly related to drug indications, contraindications, and side effects, was developed. The large language models also fact-checked these 20 medical statements. The results were compared against the evaluations of 3 clinical experts. The outcome measures were as follows: (1) percentage of accuracy of automated fact-checking, (2) time to complete fact-checking, and (3) a binary outcome for prompt redrafting (advising the patient to revise her prompt by naming her acne medication to address safety concerns).

**Results:**

For the scenario of a patient with acne, GPT-4o and OpenBioLLM-70B both had 86% agreement with the clinical experts’ fact-checking. The large language models did not consistently convey the urgency of discontinuing isotretinoin treatment when pregnancy is suspected. In addition, the models did not adequately convey the importance of folic acid supplementation during pregnancy. For the set of 20 medical claims, GPT-4o fact-checking had 100% agreement with that of human experts, whereas OpenBioLLM-70B had 95% agreement. OpenBioLLM-70B diverged from human experts and GPT-4o on 1 question related to pediatric use of antihistamines. The expert fact-checks took a mean time of 18 (SD 3.74) minutes, GPT-4o took 42 seconds, and OpenBioLLM-70B took 33 minutes. The GPT-4o responses for the acne scenario had some inconsistencies but zero fabrication and no obvious omissions. In contrast, OpenBioLLM-70B omitted 1 key information item needed for patient safety.

**Conclusions:**

GPT-4o can interact with patients to improve the quality and comprehensiveness of the information contained in health-related prompts. GPT-4o and OpenBioLLM-70B can conduct efficient fact-checking that is close to the level of accuracy of human experts. Human experts need to perform additional checks for accuracy and safety.

## Introduction

Generative artificial intelligence (GenAI) is a form of artificial intelligence that can generate text, images, or other data types. Tools such as ChatGPT and Gemini offer a new opportunity for patients and clinicians to access health information [1].

There is evidence that patients are willing to use ChatGPT for self-diagnosis [2]. Clinicians are willing to use ChatGPT for various tasks in practice [3,4]. In some cases, GenAI has suggested diagnoses that have eluded clinicians [5]. However, GenAI can provide misleading and factually incorrect information authoritatively without indicating that it is unreliable [6,7].

Clinicians might be able to distinguish factually correct from incorrect GenAI responses in their areas of expertise but not in topics in which they only have general knowledge, whereas patients will always need support to fact-check GenAI responses. There is a need for improved tools to support clinicians and patients in this process.

The role of GenAI may extend beyond answering users’ health care prompts. GenAI may also have a role to play in the subsequent fact-checking of these responses. This additional layer of “self-checking” may bolster the accuracy of responses. There is scant evidence on the ability of GenAI to perform this function. There is also scant literature describing the manner in which a patient might interact with GenAI to accomplish this in a real-world context.

Ni et al [8] evaluated the accuracy of large language models (LLMs), a prominent form of GenAI, in fact-checking public health statements. They found that the accuracy ranged from 66% to 90% depending on the type of prompt and the incorporation of relevant documents into the LLM system.

Zarharan et al [9] compared the accuracy of a number of LLMs for fact-checking public health statements. When zero-shot prompting was used, the most accurate model was GPT-4. Zero-shot prompting means that the user does not input any relevant examples from which the LLM could learn what constitutes a satisfactory response. In contrast, when the user provided one or more examples from which the model could learn (known as single- or few-shot prompting), open-source models achieved a level of accuracy that was comparable to that of the proprietary GPT-4 model. The highest accuracy was achieved by Vicuna-13B and Mistral-7B after undergoing fine-tuning, a process that adjusts some of the LLM’s weights for improved performance in a specific task. These models achieved 68.5% and 72.0% *F*_1_-scores, respectively.

LLMs may also have a role to play in patient portals. A patient portal is a mechanism whereby patients can communicate with health care providers via the electronic health record. Chen et al [10] used an LLM to draft responses to simulated patient messages (N=156) in a patient portal in a radiation oncology service at a tertiary care hospital. The LLMs’ responses were compared to responses generated by human experts. The human responses were shorter (34 vs 169 words). The LLM drafts posed a risk of severe harm in 7.1% of responses and a risk of death in 0.6% of responses. These risks were generally due to underestimating or failing to convey the acuity of a clinical scenario.

An advanced form of LLM is known as a reasoning model. Reasoning models break a prompt into smaller tasks and incrementally derive a conclusion. Vladika et al [11] used a reasoning model to verify medical statements. For each statement, an LLM generated up to 5 new questions in response. These questions were then answered using the model’s internal knowledge combined with a web search. Subsequently, another LLM integrated the responses to these questions and used this information to fact-check the original statement.

This study had 2 aims. The first was to evaluate the accuracy and efficiency of automated fact-checking by 2 LLMs. We compared the LLMs’ responses to those of human experts who searched for evidence sources. The second aim was to illustrate a process through which a patient might interact with an LLM to craft a prompt with appropriate information to support patient safety. We chose to use the following LLMs: GPT-4o (OpenAI), a commonly used framework, and OpenBioLLM-70B, an open-source model that performs strongly on biomedical benchmarks [12].

## Methods

### Patient Involvement

To ensure realism and relevance for patients, we adopted a patient-centered research approach. Our patient coauthor experienced acne in her late 20s and was prescribed treatments. She was involved in developing the clinical scenario and the initial prompt that was the basis for the evaluation.

### Ethical Considerations

No ethics approval was required for this work as it did not involve data obtained from human participants, in accordance with the policies of the Dartmouth Health Institutional Review Board.

### Scenario

**Figure 1.**
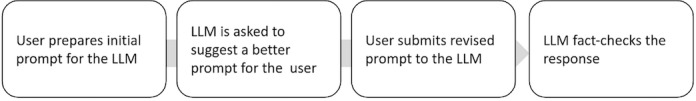
Prompt improvement and fact-checking process that can be undertaken by a patient using a large language model (LLM).

On the basis of our patient author’s experience, we proposed a query from a hypothetical patient, equivalent to a “prompt” to be given to LLMs. A hypothetical patient aged 23 years is taking isotretinoin to treat her acne. She is concerned she might be pregnant but has not had a pregnancy test. She asks an LLM for advice. Her first prompt (Textbox 1) does not specify isotretinoin. This is the first step in the process in Figure 1. This scenario contributes to both aims of this study. The LLMs generate responses that can serve as material for fact-checking (aim 1), whereas the scenario also illustrates a realistic manner in which a patient might interact with LLMs (aim 2).

Textbox 1. **Initial large language model prompt from a hypothetical patient requesting guidance on acne treatment in the context of possible pregnancy.**

“I’m a 23-year-old woman. I think I might be pregnant. I’m pretty healthy, I just take acne capsules, otherwise I’m not on any medicines. What should I do?”

### Automated Prompt Improvement and Fact-Checking Process

When patients craft a prompt for an LLM, they may fail to convey critical aspects of their condition or medical history, leading to suboptimal or even harmful responses from an LLM. To address this, we developed a redrafting prompt (Textbox 2) asking the LLMs to suggest ways to improve the patient’s initial prompt, such as by including details of medication or symptoms. This is step 2 of the process shown in Figure 1. The LLM then suggests ways to refine the patient’s initial prompt. The redrafting prompt can constrain the length of the LLM’s response to avoid overwhelming the user, for example, “Please list the three most important items of information” to include. In our approach, we did not constrain the LLM. The LLM persona for the redrafting prompt (“You’re a medical expert”) was informed by a research study by Pal et al [13].

Textbox 2. **Redrafting prompt: hypothetical patient requests guidance from a large language model to refine her original prompt regarding acne treatment in the context of possible pregnancy.**

“You’re a medical expert. Consider the statement below from a patient. Please state what additional information you would want from the patient in order to make a good decision.”

As part of this process, a patient author (OD) reviewed the LLM responses. The patient author flagged the potential harm of drug treatment as concerning; hence, our hypothetical patient added the name of her acne treatment to her revised prompt but did not make any further changes (step 3 in Figure 1).

Two LLMs, GPT-4o and OpenBioLLM-70B, generated responses for each prompt. GPT-4o was accessed via the ChatGPT web portal. OpenBioLLM-70B was deployed on Google Colab using an NVIDIA A100 graphics processing unit. The automated prompt improvement and fact-checking process is summarized in Figure 1. The prompts and the exact responses were dated and stored so that we could apply and compare the 2 fact-checking methods. The prompts were sent to GPT-4o and OpenBioLLM-70B on September 16, 2024, and January 6, 2025, respectively.

### LLM Fact-Checking

As part of this scenario, we imagined that the patient also wanted to fact-check the response to the revised prompt but was unsure about how to accomplish this task. We outlined a process through which the patient can prompt an LLM to conduct an automated fact-check of the previous response. This is compared against a manual method that we designed, which, although unlikely to be routinely used by patients or clinicians, provides a benchmark against which to compare GenAI fact-checking.

The LLM fact-check involves 2 separate prompts. The first prompt checks factuality, whereas the second prompt asks the LLM to identify inconsistencies and contradictions. These prompts are complementary. These 2 automated fact-checking prompts are shown in Textbox 3. The phrasing of the fact-checking prompt was informed by the research by Zhang et al [14].

Textbox 3. **Prompts for fact-check and consistency check: hypothetical patient asks a large language model to check the factuality and consistency of its previous responses.**

**Fact-check**

“You’re a medical expert. Evaluate the truthfulness of the statement below. Consider your sources, context and date while assessing. To answer return ‘Final Answer: {verdict}, {reason}.’ You must respond with a valid verdict: (‘false,’ ‘mostly-false,’ ‘half-true,’ or ‘true’) or ‘uncertain,’ providing reasoning and citing sources by providing the domain of pertinent search results. Here is the statement to check:”

**Consistency check**

“You’re a medical expert. Please identify any inconsistencies or contradictions in the following statement:”

### “Gold-Standard” Comparator: Fact-Checking by Human Experts

The manual fact-checking process consisted of 6 steps. To remove potential bias, one judge (author PR) completed manual fact-checking before the LLM fact-checking. The other 2 judges did not have access to the LLMs’ fact-checking responses. All 3 judges are pharmacists, and all conducted fact-checking independently. The fact-checking process steps were as follows:

- Step 1—identify information claims in the response. A claim is a discrete phrase containing relevant information. Each claim was tabulated (Multimedia Appendix 1). A sentence might contain multiple claims. For example, the response to the initial prompt referred to “blood tests, urine tests, and possibly an ultrasound.” These 3 tests were classified as 3 separate claims.
- Step 2—determine the accuracy of each claim. This can be done in several ways; for example, a simple binary classification (true or false) can be used. We adapted the approach by Quelle and Bovet [15] and used 5 categories: true, mostly true, mostly false, false, and uncertain.
- Step 3—identify contradictory or inconsistent claims.
- Step 4—identify claims that may pose clinical risk. This may comprise ambiguous, misleading, or omitted information relevant to patient safety.
- Step 5—assess the quality of the evidence that supports the response claims. This involved online searches using Google and PubMed to check the veracity of each claim. Online information sources were considered eligible if the source was judged to be credible and evidence-based. This assessment was based on the experts’ pharmacology training and interpretation of medical evidence using criteria such as sample size and study design [16].
- Step 6—assess comprehensiveness by reviewing cited sources and determining whether critical pieces of information were omitted.

### Evaluating the Generalizability of LLM Fact-Checking

As part of aim 1, we evaluated the generalizability of LLMs’ ability to fact-check health care claims. The 2 LLMs assessed the factuality of 20 clinical claims. Independently, the 3 expert reviewers assessed the same claims.

The 20 claims are shown in Multimedia Appendix 1. To achieve a balanced dataset, 10 claims were considered to be true by the human judges, and 10 were false. A blend of true and false claims enabled the calculation of a true positive rate and a true negative rate. For example, a true claim was “Mercaptopurine is indicated for maintenance of remission of Crohn’s Disease.” A false claim was “Trifluoperazine is safe in patients with phaeochromocytoma.” The claims addressed a variety of topics: clinical indications, contraindications, treatment duration, side effects of treatment, and drug interactions.

We used the following prompt for the LLMs:

You’re a medical expert. Please assess the strength of evidence for the health claim below. Consider your sources, context and date while assessing. To answer return “Final Answer: {verdict}, {reason}.” You must respond with a valid verdict: (“True,” “False”). Here is the claim to check:

This prompt was administered to both LLMs for each of the 20 claims.

### GenAI Research

A methodological checklist for GenAI research has been developed by Sallam et al [17]. This checklist can be found in Multimedia Appendix 2.

## Results

The first task for the LLMs was to offer guidance to help redraft the user’s initial prompt (second step of Figure 1). ChatGPT’s guidance for redrafting (Multimedia Appendix 1) addressed 7 topics. Crucially, GPT-4o requested more specific details on the patient’s acne medication in light of the potential harm during pregnancy. In general, GPT-4o adopted a patient-centered approach, requesting information on the user’s support system and lifestyle factors. GPT-4o’s response was extensive and could have led to a major revision of the prompt. However, the prime concern of the patient was the potential harm from acne medication, so she revised her prompt by naming the medication.

The revised prompt submitted to the LLMs was as follows:

I’m a 23-year-old woman. I think I might be pregnant. I’m pretty healthy, I just take isotretinoin for acne otherwise I’m not on any medicines. What should I do?

In contrast, OpenBioLLM-70B’s response to the redrafting prompt is shown in Multimedia Appendix 1. This response was shorter than the GPT-4o response (75 vs 279 words, respectively). OpenBioLLM-70B mentioned 6 topics, such as a missed period, home pregnancy test, and contraceptive use. However, OpenBioLLM-70B did not mention the risk of acne medication during pregnancy and did not ask the patient to provide more information on her medication. Hence, in this redrafting task, GPT-4o was superior to OpenBioLLM-70B in terms of patient safety.

After the prompt redrafting step, the revised prompt was submitted to the 2 LLMs. The LLMs both generated a response to the revised prompt. The LLMs were then prompted to fact-check the GPT-4o response as this contained more detail on the safe use of acne medication.

Table 1 shows the results of comparing human expert and automated fact-checking. The complete assessment is provided in Table S1 in Multimedia Appendix 1. The experts followed the manual fact-checking process and identified 16 claims to verify. Of the 16 claims, the experts deemed 13 (81.3%) to be true, and 3 (18.8%) were classified as mostly true. This manual process (including the search for sources and specifying citations in the table of claims) took 18 minutes (full results are shown in Multimedia Appendix 1).

**Table 1.** Results of fact-checking by GPT-4o, OpenBioLLM-70B, and human experts for isotretinoin treatment of acne in the context of possible pregnancy.

	Human experts^a^	GPT-4o	OpenBioLLM-70B
Number of claims identified	16	13	13
Number of true claims	13	13	13
Number of mostly true claims	3	0	0
Number of false claims	0	0	0
Number of uncertain claims	0	0	0
Time taken to review (mean and SD shown for the 3 expert reviewers)	18 (3.74) min	42 s	33 min, 21 s

a The following information sources were used as references in the manual search by the human experts: the National Health Service, the Centers for Disease Control and Prevention, the Health Service Executive, the National Institutes of Health, the Mayo Clinic, *PLOS One*, the National Organization for Rare Disorders, the American Pregnancy Association, and the American College of Obstetricians and Gynecologists.

By comparison, GPT-4o identified 13 claims to verify and deemed all these claims to be true. This process took 42 seconds. The experts disagreed with the LLM classification of the claim about the use of folic acid because the response understated the importance of taking folic acid during pregnancy. Disagreement about the discontinuation of isotretinoin occurred for this response. The experts believed that isotretinoin treatment should be paused immediately without waiting for the results of a pregnancy test or a clinical visit given the risk involved. The response from the LLM was inconsistent; a section early in the response recommended stopping isotretinoin after a positive pregnancy test, whereas the conclusion recommended immediate cessation of isotretinoin. This inconsistency was not detected by the consistency check (Textbox 3).

Multimedia Appendix 1 shows ChatGPT’s response to the fact-check for consistency. The LLM suggested 4 improvements: the use of more sensitive pregnancy tests, safer treatments for acne, mental health support, and follow-up monitoring for maternal and fetal health. In contrast, the experts focused primarily on the urgency of discontinuing isotretinoin treatment and seeking advice from a clinician who could devise a comprehensive care plan in cooperation with the patient.

As part of aim 1, our study also tested the generalizability of LLMs’ fact-checking capability. The 20 medical statements under scrutiny are shown in Table S2 in Multimedia Appendix 1. GPT-4o had 100% agreement with human experts (Table S3 in Multimedia Appendix 1), whereas OpenBioLLM-70B had 95% agreement (Table S4 in Multimedia Appendix 1). For example, both LLMs found the following statement to be true:

Combined hormonal contraceptives should not be used in patients with acute porphyrias or gallstones.

Both LLMs correctly deemed the following statement to be false:

Sumatriptan is used to treat dizziness and drowsiness.

One question related to chlorphenamine, an antihistamine drug. In its response, OpenBioLLM-70B incorrectly stated the following:

False, The evidence does not support the claim that chlorphenamine can relieve the symptoms of allergy in children as young as 1 month.

In contrast, GPT-4o and the 3 human experts asserted that there was evidence to support the use of chlorphenamine in this age group. GPT-4o mentioned the following:

According to reputable sources, including the British National Formulary for Children (BNFc), chlorphenamine can be used in infants as young as 1 month old, albeit with adjusted dosing and under medical supervision. Its use in this age group is typically reserved for specific indications due to concerns about sedation and anticholinergic side effects. Still, the evidence supports its effectiveness and safety when properly dosed and monitored.

The complete LLM responses to fact-checking of these 20 medical statements can be found in Tables S3 and S4 in Multimedia Appendix 1.

## Discussion

### Principal Findings

This scenario-based study demonstrates that an LLM can become a valuable source of evidence-based information by being prompted to undertake self-checking processes. The responses were checked by 3 experts and found to be fact-based; there was no fabrication or essential omissions. However, the LLMs did not consistently recommend immediately stopping isotretinoin or sufficiently guide the patient to take folic acid. A clinician could have corrected these errors, but a patient could have been misled.

By using methods in which LLMs are asked to revise the quality of prompts and undertake fact-checking for truthfulness and consistency, we showed that LLM responses could be improved to the point where the results showed an 86% agreement with those of experts undertaking verification. There are efficiency implications as the GPT-4o process was 24 times faster than the check conducted by the experts. Such capability and speed are superior by far to those of a nonexpert. The use of OpenBioLLM-70B was slower than that of the human experts, but the use of infrastructure on Google Colab was not optimized for quick model serving. OpenBioLLM-70B deployment could, in principle, be accelerated to match the latency of GPT-4o.

### Results in the Context of Similar Studies

Our study aligns with recent evidence that LLMs can play a helpful role in fact-checking in various domains [18]. LLMs are more accurate on health-related questions when scientific consensus exists but are less able to respond when the evidence is controversial, ambiguous, or recently published [19]. Kusunose et al [20] found that GPT-3.5 achieved 80% accuracy for questions about hypertension guidelines, but the accuracy dropped to 36% when the evidence was weaker. LLMs can support shared decision-making by highlighting for clinicians the questions that patients are most likely to ask and providing written information in accessible ways to patients [21]. Standardized datasets have been developed to measure the factuality of LLMs in domains such as health care [22]. However, expert-crafted questions may differ significantly from patients’ questions in terms of clarity and inclusion of relevant information. Thus, it is important to involve patients in this process of LLM validation.

### Unanswered Questions and Future Research

Despite our results, we do not recommend using LLMs in routine clinical practice without clinician verification. However, as patients will inevitably use LLMs and find flawed information that will be misinterpreted [23], clinicians must find methods to adapt to LLMs. Future research needs to further evaluate the capability of LLMs to self-improve and self-check comparing the responses to realistic benchmarks. As LLMs are based on stochastic algorithms, future research can investigate mechanisms to integrate LLMs with rule-based logic systems to mitigate inaccuracy and improve fact-checking. Bangerter et al [24] found that a hybrid approach to fact-checking combining LLMs and fuzzy logic increased reliability, but this was not applied to health care claims.

Further research is also needed on the use of LLM agents. Agents can engage in back-and-forth with patients to ensure an appropriate level of detail for evidence-based decision-making. Agents can then craft an optimal prompt and deliver this to a subject matter expert LLM. Research is also needed into optimal methods of keeping LLMs up-to-date with new evidence. This could be a blend of fine-tuning some parameters of the LLM and giving the LLM access to new documents in a technique known as retrieval-augmented generation. Another fruitful area for research is likely to be multimodal artificial intelligence models, for example, integrating medical imaging, data tables (eg, trends of blood pressure and cholesterol measurements), and natural language input.

A multidisciplinary approach is needed to ensure the safe use of LLMs. Haltaufderheide and Ranisch [25] argue that the degree of acceptable human oversight for LLMs in health care varies depending on the potential for harm. Han et al [26] found that subtle modification of approximately 1% of an LLM’s parameters can cause it to learn incorrect biomedical facts. There is a risk of malicious actors exploiting this vulnerability.

### Strengths and Weaknesses of This Method

A strength of this study was the use of an actual clinical question: a concerning situation for many who have acne and are sexually active. The clinical scenario was co-designed with our patient coauthor, who also guided the development of our methods and interpretations of the responses. All prompts and LLM responses were dated and archived to ensure transparency and reproducibility. We demonstrated how to increase the ability of LLMs to rapidly deliver high-quality information by prompting GenAI systems to self-improve and self-evaluate. This mimics a process whereby a clinician asks a patient to provide additional information. Given the inevitable use of LLMs as a source of health care information, these prompt revision and fact-checking methods are relevant to a wide audience. We used a panel of 3 judges to bolster the validity of the evaluation. We tested the generalizability of fact-checking by using 20 claims on disparate health care topics (Multimedia Appendix 1).

Our study has a number of limitations. Only 1 detailed clinical scenario was developed related to acne management. Many other clinical scenarios should be evaluated to improve our understanding of the capabilities of LLMs. Our study used 2 LLMs for fact-checking, but new LLMs are being developed that may improve the accuracy of fact-checking. There may be scope to improve performance by using a specialized LLM for fact-checking and another LLM to respond directly to patients’ prompts. A rigorous comparison of prompt engineering techniques for fact-checking was not conducted in our study. Furthermore, although our patient author (OD) improved the realism of our scenario, there may have been some Hawthorne effect, whereby behavior changes when a person knows they are under observation. Patients may interact differently with LLMs in a natural setting. Finally, fact-checking, even by experts, is imperfect. Clinical guidelines and high-quality scientific literature reviews often have inconsistencies, and interpretations of evidence may differ [27,28]. This was mitigated by the use of 3 human experts for fact-checking, but there is still potential for inaccuracy.

### Broader Implications

Scientific processes rely on self-correction, such as peer review, which, although fallible, over time serves to separate fact from fiction. Given that patients will turn to LLMs, regulators could set accuracy thresholds for GenAI [29]. It may be feasible to build in prompt improvement and fact-checking when health care questions are submitted and withhold responses if questions are unclear [30] or if there is insufficient scientific consensus.

## Supplementary material

10.2196/68223 Multimedia Appendix 1 Prompts and large language model responses.

10.2196/68223 Multimedia Appendix 2 Methodological checklist.

## Abbreviations:

GenAI: generative artificial intelligence
LLM: large language model

## References

[1] Mirza FN, Tang OY, Connolly ID (2024). Using ChatGPT to facilitate truly informed medical consent. NEJM AI.

[2] Shahsavar Y, Choudhury A (2023). User intentions to use ChatGPT for self-diagnosis and health-related purposes: cross-sectional survey study. JMIR Hum Factors.

[3] Kisvarday S, Yan A, Yarahuan J (2024). ChatGPT use among pediatric health care providers: cross-sectional survey study. JMIR Form Res.

[4] Blease CR, Locher C, Gaab J, Hägglund M, Mandl KD (2024). Generative artificial intelligence in primary care: an online survey of UK general practitioners. BMJ Health Care Inform.

[5] Topol EJ (2024). Toward the eradication of medical diagnostic errors. Science.

[6] Cao JJ, Kwon DH, Ghaziani TT (2023). Accuracy of information provided by ChatGPT regarding liver cancer surveillance and diagnosis. AJR Am J Roentgenol.

[7] Rao A, Pang M, Kim J (2023). Assessing the utility of ChatGPT throughout the entire clinical workflow: development and usability study. J Med Internet Res.

[8] Ni Z, Qian Y, Chen S, Jaulent MC, Bousquet C (2024). Scientific evidence and specific context: leveraging large language models for health fact-checking. Online Inf Rev.

[9] Zarharan M, Wullschleger P, Pilehvar MT, Foster J, Kia BB (2024). Tell me why: explainable public health fact-checking with large language models. arXiv.

[10] Chen S, Guevara M, Moningi S (2024). The effect of using a large language model to respond to patient messages. Lancet Digit Health.

[11] Vladika J, Hacajova I, Matthes F (2025). Step-by-step fact verification system for medical claims with explainable reasoning. arXiv.

[12] Dorfner FJ, Dada A, Busch F (2024). Biomedical large languages models seem not to be superior to generalist models on unseen medical data. arXiv.

[13] Pal A, Umapathi LK, Sankarasubbu M Med-HALT: medical domain hallucination test for large language models. https://aclanthology.org/2023.conll-1.21.pdf.

[14] Zhang C, Yang R, Zhang Z (2024). Atomic calibration of LLMs in long-form generations.

[15] Quelle D, Bovet A (2024). The perils and promises of fact-checking with large language models. Front Artif Intell.

[16] Atkins D, Best D, Briss PA (2004). Grading quality of evidence and strength of recommendations. BMJ.

[17] Sallam M, Barakat M, Sallam M (2024). A preliminary checklist (METRICS) to standardize the design and reporting of studies on generative artificial intelligence-based models in health care education and practice: development study involving a literature review. Interact J Med Res.

[18] Augenstein I, Baldwin T, Cha M (2024). Factuality challenges in the era of large language models and opportunities for fact-checking. Nat Mach Intell.

[19] Srivastava B (2021). Did chatbots miss their “Apollo Moment”? Potential, gaps, and lessons from using collaboration assistants during COVID-19. Patterns (N Y).

[20] Kusunose K, Kashima S, Sata M (2023). Evaluation of the accuracy of ChatGPT in answering clinical questions on the Japanese Society of Hypertension Guidelines. Circ J.

[21] Elwyn G, Ryan P, Blumkin D, Weeks WB (2024). Meet generative AI… your new shared decision-making assistant. BMJ Evid Based Med.

[22] Lin S, Hilton J, Evans O TruthfulQA: measuring how models mimic human falsehoods. https://aclanthology.org/2022.acl-long.229.pdf.

[23] Small WR, Wiesenfeld B, Brandfield-Harvey B (2024). Large language model-based responses to patients' in-basket messages. JAMA Netw Open.

[24] Bangerter ML, Fenza G, Furno D (2024). A hybrid framework integrating LLM and ANFIS for explainable fact-checking. IEEE Trans Fuzzy Syst.

[25] Haltaufderheide J, Ranisch R (2024). The ethics of ChatGPT in medicine and healthcare: a systematic review on Large Language Models (LLMs). NPJ Digit Med.

[26] Han T, Nebelung S, Khader F (2024). Medical large language models are susceptible to targeted misinformation attacks. NPJ Digit Med.

[27] O’Connell NE, Cook CE, Wand BM, Ward SP (2016). Clinical guidelines for low back pain: a critical review of consensus and inconsistencies across three major guidelines. Best Pract Res Clin Rheumatol.

[28] Cotie LM, Vanzella LM, Pakosh M, Ghisi GL (2024). A systematic review of clinical practice guidelines and consensus statements for cardiac rehabilitation delivery: consensus, divergence, and important knowledge gaps. Can J Cardiol.

[29] Liu S, Wright AP, Mccoy AB (2024). Using large language model to guide patients to create efficient and comprehensive clinical care message. J Am Med Inform Assoc.

[30] Han T, Adams LC, Bressem KK, Busch F, Nebelung S, Truhn D (2024). Comparative analysis of multimodal large language model performance on clinical vignette questions. JAMA.

