# Supplementary material for: Fact-Checking Large Language Model Responses to a Health Care Prompt: Comparative Study
Source: JMIR Form Res. 2026 Apr 15;10:e68223. doi: 10.2196/68223 (PMC13082570; doi:10.2196/68223)
Supplement: Multimedia Appendix 1 [file formative-v10-e68223-s001.doc]

Fact-checking large language model responses to a healthcare prompt: A comparison study.

[**Table A1 Results of Fact-Checking by ChatGPT, OpenBioLLM-70b, and by Human Experts for isotretinoin treatment of acne in the context of possible pregnancy 1**](#_fqleosaxhj1x)

[**Table A2: Manual fact-check of twenty healthcare claims relating to a diverse set of clinical scenarios 2**](#_n35djnd50xfn)

[**Table A3: ChatGPT-4o fact-check of twenty healthcare claims relating to a diverse set of clinical scenarios. 3**](#_oq68e8luj74b)

[**Table A4: OpenBioLLM-70b fact-check of twenty healthcare claims relating to a diverse set of clinical scenarios. 7**](#_vb6u6xj1497m)

[**Appendix A5: ChatGPT-4o Response to initial prompt relating to isotretinoin treatment in the context of possible pregnancy. 9**](#_s57joprddxsr)

[**Appendix A6: ChatGPT-4o response to Redrafting Prompt to elicit additional information from a patient considering isotretinoin treatment in the context of possible pregnancy 10**](#_u3p1mgw0mub4)

[**Appendix A7: ChatGPT-4o Response to Patient’s Revised Prompt relating to isotretinoin treatment in the context of possible pregnancy. 12**](#_8m3y7o8o77at)

[**Appendix A8: ChatGPT-4o performs a consistency check on its prior response relating to isotretinoin use in the context of possible pregnancy. 13**](#_kt9e6gpwplqt)

[**Appendix A9: OpenBioLLM-70b response to initial prompt relating to isotretinoin use in the context of possible pregnancy. 14**](#_551zvaik2yyl)

[**Appendix A10: OpenBioLLM-70b response to a Redrafting Prompt to elicit additional information from a patient considering isotretinoin treatment in the context of possible pregnancy 14**](#_27tsiii8xkqs)

[**Appendix A11: OpenBioLLM-70b response to patient’s Revised Prompt relating to isotretinoin treatment in the context of possible pregnancy. 15**](#_grtyen8z61v0)

[**Appendix A12: OpenBioLLM-70b performs a consistency check on ChatGPT’s response to a patient considering isotretinoin treatment in the context of possible pregnancy. 15**](#_gamlh3o2k64m)

[**Table A13 Author Discipline, Institution, Location and Contribution. 15**](#_a28r5gkozfdd)

**Multimedia Appendix:**

# Table A1 Results of Fact-Checking by ChatGPT, OpenBioLLM-70b, and by Human Experts for isotretinoin treatment of acne in the context of possible pregnancy

|  | **Claim** | **Expert human assessment** | **ChatGPT** | **OpenBioLLM-70b** |
| --- | --- | --- | --- | --- |
| **1** | Isotretinoin is known to be highly teratogenic, meaning it can cause serious birth defects. | True | True | True |
| **2** | Use a home pregnancy test after a missed period for the most accurate result. | True | True | True |
| **3** | Regardless of the home test result, schedule an appointment with your healthcare provider to confirm the pregnancy with a blood test and clinical examination. | True | True | True |
| **4** | If pregnancy is confirmed, you should immediately stop taking isotretinoin. This medication is known to cause severe birth defects and is contraindicated during pregnancy. | Mostly true | True | True |
| **5** | Your healthcare provider will provide guidance on how to safely discontinue isotretinoin and discuss alternative treatments for acne if necessary. | True | True | True |
| **6** | Schedule an appointment with a healthcare provider for early prenatal care to monitor your health and the development of the baby. | True | True | True |
| **7** | Provide your healthcare provider with a full history of your isotretinoin use, including the duration and dosage. | True | True | True |
| **8** | There are safer options for managing acne during pregnancy, such as topical treatments that are not known to harm the developing fetus. | True | True | True |
| **9** | Some acne treatments are not recommended during pregnancy, so it's important to choose options that are safe. | True | True | True |
| **10** | Focus on a balanced diet and consider taking prenatal vitamins, particularly those with folic acid, to support a healthy pregnancy. | Mostly true | True | True |
| **11** | Refrain from alcohol, tobacco, and illicit drugs. Limit caffeine intake and consult your healthcare provider before taking any over-the-counter medications or supplements. | True | True | True |
| **12** | Educational resources and support groups for expecting mothers can provide valuable information and support during your pregnancy. | True | True | True |
| **13** | If you have concerns about your mental health or the impact of pregnancy on your well-being, discuss these with your healthcare provider. | True | True | True |

# Table A2: Manual fact-check of twenty healthcare claims relating to a diverse set of clinical scenarios

|  | **Claim** | **Human assessment** |
| --- | --- | --- |
| **1** | Isotretinoin should be discontinued during pregnancy | True |
| **2** | Antiplatelet drugs can help to prevent stroke. | True |
| **3** | Oxytocin can be used to induce labour. | True |
| **4** | Mercaptopurine is indicated for maintenance of remission of Crohn's Disease. | True |
| **5** | Salbutamol is a first-line treatment for asthma. | True |
| **6** | Beta blockers are often indicated after myocardial infarction. | True |
| **7** | Combined hormonal contraceptives should not be used in patients with acute porphyrias or gallstones. | True |
| **8** | There is a risk of infection after total knee replacement surgery. | True |
| **9** | Some patients with heart failure will use an implantable cardioverter defibrillator and continue to take medication | True |
| **10** | Chlorphenamine can relieve the symptoms of allergy in children as young as 1 month. | True |
| **11** | Methotrexate is first-line treatment for a headache | False |
| **12** | Mercaptamine is indicated for treatment of leukemia. | False |
| **13** | Cyclizine is indicated for the treatment of Parkinson's Disease. | False |
| **14** | Tetracycline is indicated for the treatment of agranulocytosis | False |
| **15** | Triptorelin is generally used for three years in the treatment of endometriosis. | False |
| **16** | Mefenamic acid is used to lower blood pH. | False |
| **17** | Naproxen is used to treat gastrointestinal ulcers. | False |
| **18** | St John's Wort does not reduce the effectiveness of oral contraceptives. | False |
| **19** | Trifluoperazine is safe in patients with phaeochromocytoma. | False |
| **20** | Sumatriptan is used to treat dizziness and drowsiness. | False |

# Table A3: ChatGPT-4o fact-check of twenty healthcare claims relating to a diverse set of clinical scenarios.

| No. | ChatGPT-4o response |
| --- | --- |
| 1 | Final Answer: **True**, **because there is strong and consistent evidence that isotretinoin is highly teratogenic (causes birth defects) and poses serious risks to fetal development. It is classified as Pregnancy Category X by the FDA, indicating that the risks in pregnancy clearly outweigh any potential benefits. Multiple studies and decades of clinical experience have confirmed that isotretinoin exposure during pregnancy is associated with a high risk of severe congenital malformations, miscarriage, and developmental abnormalities. Therefore, discontinuation during pregnancy is not only recommended but mandated in many guidelines, including those from the FDA and major dermatological associations.** |
| 2 | Final Answer: **True**, **because there is strong, high-quality evidence from randomized controlled trials and meta-analyses that antiplatelet drugs, such as aspirin and clopidogrel, reduce the risk of ischemic stroke, particularly in patients with a history of stroke, transient ischemic attack (TIA), or atherosclerotic cardiovascular disease. Guidelines from major health organizations, including the American Heart Association (AHA) and European Stroke Organisation, support their use for secondary stroke prevention. The benefit in primary prevention is more selective and based on individual risk factors, but the overall evidence supports their efficacy in stroke prevention in appropriate populations.** |
| 3 | Final Answer: **True**, **because there is strong and longstanding evidence from clinical trials, guidelines, and routine obstetric practice that oxytocin is effective for inducing labor. Oxytocin, a synthetic form of the natural hormone, stimulates uterine contractions and is commonly used in hospital settings to initiate or augment labor. It is recommended by authoritative bodies such as the World Health Organization (WHO), the American College of Obstetricians and Gynecologists (ACOG), and the National Institute for Health and Care Excellence (NICE). Its use is supported by decades of clinical data demonstrating its efficacy and safety when used with appropriate monitoring.** |
| 4 | Final Answer: True, because there is strong evidence and clinical guideline support for the use of mercaptopurine (6-mercaptopurine, or 6-MP) as a maintenance therapy in Crohn’s disease. It is an immunosuppressive thiopurine agent that helps maintain remission by reducing immune system activity and inflammation. Multiple randomized controlled trials and long-term observational studies have demonstrated its efficacy in maintaining remission, especially after corticosteroid-induced remission. Guidelines from organizations such as the American College of Gastroenterology (ACG), European Crohn’s and Colitis Organisation (ECCO), and British Society of Gastroenterology (BSG) include mercaptopurine as a recommended option for maintenance therapy in patients with Crohn’s disease. |
| 5 | Final Answer: **True**, **because salbutamol (also known as albuterol) is a short-acting beta-2 agonist (SABA) that provides rapid bronchodilation and is widely recognized as a first-line treatment for the relief of acute asthma symptoms. It is included in national and international asthma management guidelines, such as those from the Global Initiative for Asthma (GINA), as a rescue medication for immediate symptom relief. While recent guidelines emphasize the need for concurrent anti-inflammatory treatment (e.g., inhaled corticosteroids) to manage underlying inflammation, salbutamol remains a cornerstone of acute symptom management due to its rapid onset and effectiveness.** |
| 6 | Final Answer: **True**, **because there is strong and consistent evidence from randomized controlled trials and clinical guidelines that beta blockers reduce mortality and the risk of recurrent myocardial infarction (MI) when used after an acute MI. They work by decreasing myocardial oxygen demand, reducing heart rate, and preventing arrhythmias. Major clinical guidelines from the American Heart Association (AHA), American College of Cardiology (ACC), and European Society of Cardiology (ESC) recommend beta blockers as part of standard post-MI therapy, particularly in patients with reduced left ventricular function or heart failure. Their benefits are most pronounced when initiated early and continued long-term in appropriate patients.** |
| 7 | Final Answer: **True**, **because there is strong clinical guidance and mechanistic rationale indicating that combined hormonal contraceptives (CHCs) are contraindicated or should be used with caution in patients with acute porphyrias and gallstones. In acute hepatic porphyrias (e.g., acute intermittent porphyria), estrogen-containing contraceptives can trigger attacks by inducing hepatic cytochrome P450 enzymes involved in porphyrin metabolism. Similarly, CHCs can increase the risk of gallstone formation by altering bile composition and motility, and may exacerbate pre-existing gallbladder disease. Reputable sources including the World Health Organization (WHO) Medical Eligibility Criteria and various hepatology and gynecology guidelines advise against CHC use in these conditions.** |
| 8 | Final Answer: **True**, **because there is well-documented and consistently observed evidence that infection is a recognized complication of total knee replacement (TKR) surgery. The risk of periprosthetic joint infection (PJI), although relatively low (typically around 1–2%), is a serious concern due to its potential for significant morbidity, need for revision surgery, and long-term antibiotic therapy. This risk is acknowledged in clinical guidelines from orthopedic and infectious disease societies, including the American Academy of Orthopaedic Surgeons (AAOS) and the CDC. Factors such as diabetes, obesity, immunosuppression, and surgical technique influence the risk level. Preventive strategies are routinely implemented to mitigate this risk.** |
| 9 | Final Answer: **True**, **because there is strong evidence from randomized controlled trials and clinical guidelines that some patients with heart failure, particularly those with reduced ejection fraction (HFrEF), benefit from implantable cardioverter defibrillators (ICDs) to prevent sudden cardiac death. These devices are typically used in conjunction with ongoing pharmacologic therapy, including beta blockers, ACE inhibitors/ARBs/ARNIs, mineralocorticoid receptor antagonists, and SGLT2 inhibitors. ICDs address arrhythmic risk, while medications improve symptoms, reduce hospitalizations, and slow disease progression. This combined approach is endorsed by major cardiology societies such as the American College of Cardiology (ACC), American Heart Association (AHA), and European Society of Cardiology (ESC).** |
| 10 | Final Answer: **True**, **because chlorphenamine (also known as chlorpheniramine) is an older first-generation antihistamine with documented efficacy in relieving allergy symptoms such as rhinitis, urticaria, and other histamine-mediated conditions. According to reputable sources, including the British National Formulary for Children (BNFc), chlorphenamine can be used in infants as young as 1 month old, albeit with adjusted dosing and under medical supervision. Its use in this age group is typically reserved for specific indications due to concerns about sedation and anticholinergic side effects, but the evidence supports its effectiveness and safety when properly dosed and monitored.** |
| 11 | Final Answer: **False**, **because methotrexate is not indicated for the treatment of headaches and is not considered a first-line—or any-line—therapy for this condition. Methotrexate is an antimetabolite and immunosuppressant commonly used for autoimmune diseases such as rheumatoid arthritis and psoriasis, and in certain cancers. Headaches are not among its approved or evidence-supported indications. First-line treatments for headaches depend on the type (e.g., tension-type, migraine, cluster) and typically include analgesics (like acetaminophen or NSAIDs), triptans for migraines, or preventative agents such as beta blockers or anticonvulsants. There is no strong or credible evidence supporting methotrexate for headache management.** |
| 12 | Final Answer: **False**, **because mercaptamine (also known as cysteamine) is not indicated for the treatment of leukemia. It is primarily used for the treatment of nephropathic cystinosis, a rare lysosomal storage disorder, by reducing cystine accumulation within cells. It may also have applications in certain rare metabolic or genetic conditions, but there is no robust clinical evidence or regulatory approval supporting its use in leukemia treatment. Leukemia therapies typically involve chemotherapeutic agents such as methotrexate, cytarabine, and targeted therapies depending on the leukemia subtype. Authoritative sources such as the FDA, EMA, and clinical guidelines do not list mercaptamine as a leukemia treatment.** |
| 13 | Final Answer: False, because cyclizine is not indicated for the treatment of Parkinson’s disease. Cyclizine is an antihistamine with antiemetic and anticholinergic properties, primarily used to treat nausea, vomiting, and motion sickness. While it has central nervous system effects, it does not address the underlying dopaminergic deficiency or motor symptoms characteristic of Parkinson’s disease. Approved treatments for Parkinson’s include dopaminergic agents such as levodopa, dopamine agonists, MAO-B inhibitors, and others supported by strong clinical evidence and international guidelines (e.g., NICE, AAN). Cyclizine is not supported by such evidence and may even worsen Parkinsonian symptoms due to its anticholinergic effects. |
| 14 | Final Answer: **False**, **because tetracycline is not indicated for the treatment of agranulocytosis. Agranulocytosis is a serious condition characterized by an extremely low number of neutrophils, leading to a high risk of severe infection. Management typically involves immediate discontinuation of any causative drugs, administration of broad-spectrum antibiotics if infection is suspected, and sometimes use of granulocyte colony-stimulating factors (e.g., filgrastim) to stimulate neutrophil production. Tetracycline is a broad-spectrum antibiotic primarily used for infections such as acne, respiratory tract infections, and certain zoonotic diseases, but it is not appropriate or effective for treating the underlying cause or complications of agranulocytosis.** |
| 15 | Final Answer: **False**, **because while triptorelin—a gonadotropin-releasing hormone (GnRH) agonist—is an effective treatment for endometriosis, it is **not generally used for three years** due to significant side effects associated with long-term use, particularly hypoestrogenism (e.g., bone mineral density loss, vasomotor symptoms). Clinical guidelines and drug labeling typically recommend a treatment duration of **3 to 6 months**, with some cases extending to **12 months** when combined with *add-back* therapy (low-dose estrogen and/or progestin) to mitigate side effects. Extended use beyond 12 months is uncommon and only considered in select, closely monitored cases. Thus, a general use period of three years is not supported by current evidence or practice guidelines.** |
| 16 | Final Answer: **False**, **because mefenamic acid is a nonsteroidal anti-inflammatory drug (NSAID) used primarily for the treatment of mild to moderate pain, including menstrual pain, and inflammation. It has no role in lowering blood pH. In fact, like other NSAIDs, mefenamic acid can cause metabolic disturbances such as metabolic alkalosis or acidosis in rare cases, but it is not used therapeutically to alter blood pH. Management of blood pH abnormalities (e.g., acidosis) depends on the underlying cause and may involve agents like sodium bicarbonate, not NSAIDs. There is no clinical or pharmacological evidence supporting the use of mefenamic acid for acid-base balance.** |
| 17 | Final Answer: False, because naproxen is a nonsteroidal anti-inflammatory drug (NSAID) that is actually associated with an increased risk of gastrointestinal (GI) ulcers, not their treatment. Like other NSAIDs, naproxen inhibits prostaglandin synthesis, which can compromise the protective lining of the stomach and duodenum, leading to mucosal injury, ulceration, and gastrointestinal bleeding. Clinical guidelines recommend caution or co-prescription of gastroprotective agents (e.g., proton pump inhibitors) when using NSAIDs in at-risk patients. The treatment of gastrointestinal ulcers typically involves acid-suppressing medications like PPIs (e.g., omeprazole) and addressing underlying causes such as Helicobacter pylori infection—not NSAIDs like naproxen. |
| 18 | Final Answer: **False**, **because there is strong pharmacokinetic and clinical evidence that St John's Wort (Hypericum perforatum) can reduce the effectiveness of oral contraceptives. St John's Wort is a known inducer of cytochrome P450 enzymes, particularly CYP3A4, and P-glycoprotein, which can accelerate the metabolism of estrogen and progestin components in oral contraceptives. This interaction can lead to subtherapeutic hormone levels, increasing the risk of breakthrough bleeding and unintended pregnancy. Multiple studies and drug interaction databases, as well as regulatory agencies like the FDA and EMA, warn against concurrent use without medical supervision.** |
| 19 | Final Answer: **False**, **because trifluoperazine, a typical antipsychotic in the phenothiazine class, is *not* considered safe in patients with phaeochromocytoma. Phaeochromocytoma is a catecholamine-secreting tumor, and drugs that can provoke catecholamine release or interfere with autonomic regulation—such as phenothiazines—pose a risk of precipitating hypertensive crises or arrhythmias. Trifluoperazine has known effects on the autonomic nervous system and may exacerbate blood pressure instability in these patients. Clinical guidelines and pharmacology references list pheochromocytoma as a contraindication or caution for phenothiazines, including trifluoperazine, due to the potential for serious cardiovascular complications.** |
| 20 | Final Answer: **False**, **because sumatriptan is not indicated for the treatment of dizziness or drowsiness. Sumatriptan is a selective serotonin 5-HT₁B/₁D receptor agonist used specifically for the acute treatment of migraine and cluster headaches. Its mechanism targets the cranial blood vessels and inflammatory pathways involved in migraine pathophysiology. Dizziness and drowsiness are actually listed as potential side effects of sumatriptan, not therapeutic targets. There is no evidence from clinical trials or guidelines supporting its use for managing non-headache symptoms like isolated dizziness or drowsiness.** |

# Table A4: OpenBioLLM-70b fact-check of twenty healthcare claims relating to a diverse set of clinical scenarios.

| No. | OpenBioLLM-70b response |
| --- | --- |
| 1 | Final Answer: True, Isotretinoin is a Category X drug during pregnancy, which means it is highly teratogenic and should be discontinued to avoid serious birth defects. |
| 2 | Final Answer: True, Antiplatelet drugs have been shown to be effective in preventing stroke, particularly in patients who have had a previous stroke or transient ischemic attack (TIA). |
| 3 | Final Answer: True, Oxytocin is commonly used to induce labour as it stimulates uterine contractions, which can help initiate childbirth. |
| 4 | Final Answer: True, There is evidence to support the use of mercaptopurine for the maintenance of remission in Crohn's Disease. |
| 5 | Final Answer: True, Salbutamol is a first-line treatment for asthma as it is a short-acting beta2-agonist that provides quick relief by relaxing the muscles in the airways and improving breathing. |
| 6 | Final Answer: True, Beta blockers are often indicated after myocardial infarction to reduce the risk of future heart attacks and improve survival. |
| 7 | Final Answer: True, The evidence from multiple sources, including the British National Formulary and the European Medicines Agency, indicates that combined hormonal contraceptives should not be used in patients with acute porphyrias or gallstones due to potential adverse effects. |
| 8 | Final Answer: True, There is a risk of infection after total knee replacement surgery. |
| 9 | Final Answer: True, This statement is true as some patients with heart failure may benefit from the use of an implantable cardioverter defibrillator (ICD) in addition to medication. The ICD helps to regulate the heart's rhythm and prevent life-threatening arrhythmias. |
| 10 | Final Answer: False, The evidence does not support the claim that chlorphenamine can relieve the symptoms of allergy in children as young as 1 month. |
| 11 | Final Answer: False, Methotrexate is not considered a first-line treatment for headaches. |
| 12 | Final Answer: False, There is no evidence to support the claim that Mercaptamine is indicated for the treatment of leukemia. |
| 13 | Final Answer: False, There is no evidence to support the use of Cyclizine for the treatment of Parkinson's Disease. |
| 14 | Final Answer: False, The evidence is not strong enough to support the claim that Tetracycline is indicated for the treatment of agranulocytosis. |
| 15 | Final Answer: False, The duration of treatment with triptorelin for endometriosis is typically limited to 6 months, as prolonged use can lead to bone loss and other side effects. |
| 16 | Final Answer: False, Mefenamic acid is a nonsteroidal anti-inflammatory drug (NSAID) used to relieve pain, not to lower blood pH. |
| 17 | Final Answer: False, The statement is incorrect because naproxen is a nonsteroidal anti-inflammatory drug (NSAID) that can actually increase the risk of gastrointestinal ulcers and bleeding, rather than treating them. |
| 18 | Final Answer: False, The evidence from multiple studies suggests that St John's Wort can significantly decrease the effectiveness of oral contraceptives by increasing the metabolism of estrogenic compounds. |
| 19 | Final Answer: False, The evidence is based on a single case report, which is considered low-quality evidence. |
| 20 | Final Answer: False, The statement is incorrect because Sumatriptan is not used to treat dizziness and drowsiness; it is actually used for the treatment of migraine headaches. |

# Appendix A5: ChatGPT-4o Response to initial prompt relating to isotretinoin treatment in the context of possible pregnancy.

**ChatGPT-4o Response:**

*“*If you think you might be pregnant, there are several important steps you should take to confirm your pregnancy and ensure both your health and the health of your potential baby. Here’s a comprehensive guide:

1. Confirm the Pregnancy

- Home Pregnancy Test: Use a home pregnancy test to check for pregnancy. These tests are available over-the-counter at pharmacies and are generally reliable. For the most accurate result, follow the instructions carefully and use the test after your missed period or according to the test's guidelines.

- Consult a Healthcare Provider: Regardless of the home test result, it's a good idea to schedule an appointment with a healthcare provider for a blood test and a clinical examination to confirm the pregnancy and begin prenatal care.

2. Review Your Acne Medication

- Consult Your Doctor: If you are taking acne capsules, it is crucial to discuss them with your healthcare provider. Some acne medications, such as certain oral retinoids, can be harmful during pregnancy. Your provider can help you switch to safer alternatives if you are pregnant.

- Medication Review: Provide your healthcare provider with a list of all medications and supplements you are taking to assess their safety during pregnancy.

3. Begin Prenatal Care

- Early Prenatal Care: Schedule a prenatal appointment as soon as your pregnancy is confirmed. Early prenatal care is important for monitoring your health and the development of the baby.

- Routine Tests and Screenings: Your healthcare provider will recommend tests and screenings to ensure a healthy pregnancy. These may include blood tests, urine tests, and possibly an ultrasound.

4. Adopt a Healthy Lifestyle

- Nutrition: Focus on a balanced diet rich in essential nutrients. Folate (or folic acid) is particularly important for preventing neural tube defects, so consider taking a prenatal vitamin that contains folic acid if advised by your provider.

- Avoid Harmful Substances: Avoid alcohol, tobacco, and illicit drugs. Limit caffeine intake and discuss any concerns about over-the-counter medications with your healthcare provider.

5. Address Lifestyle and Health Changes

- Exercise: Engage in regular, moderate exercise unless your healthcare provider advises otherwise. Exercise can help with overall health and pregnancy wellness.

- Avoid Stress: Manage stress through relaxation techniques, support networks, or counseling if needed.

6. Seek Guidance and Support

- Educational Resources: Consider seeking resources and support groups for expecting mothers. Many communities offer educational classes and support networks for new parents.

- Mental Health: If you have concerns about your mental health or how pregnancy might affect it, discuss these with your healthcare provider.

Sources for More Information

- American College of Obstetricians and Gynecologists (ACOG): Provides information on pregnancy care and what to expect.

- Mayo Clinic: Offers resources on early pregnancy care and healthy lifestyle choices during pregnancy.

- Centers for Disease Control and Prevention (CDC): Provides guidelines on prenatal health and pregnancy safety.

Conclusion

Confirm your pregnancy with a home test and follow up with a healthcare provider to ensure you receive appropriate prenatal care. Review any medications you're taking, especially acne treatments, with your provider to ensure they are safe for pregnancy. Adopting a healthy lifestyle and seeking early and regular prenatal care will help support a healthy pregnancy.*”*

# Appendix A6: ChatGPT-4o response to Redrafting Prompt to elicit additional information from a patient considering isotretinoin treatment in the context of possible pregnancy

**Response from ChatGPT-4o:**

“To provide a well-informed response to the patient’s situation, I would want to gather additional information in several key areas:

**1. Symptoms and Signs:**

- What symptoms are you experiencing that make you think you might be pregnant (e.g., missed period, nausea, breast tenderness)?

- Have you taken a home pregnancy test? If so, what were the results?

**2. Menstrual History:**

- When was your last menstrual period, and how regular is your menstrual cycle?

- Have there been any recent changes to your menstrual cycle?

**3. Sexual History:**

- When was your last sexual encounter, and what form of contraception (if any) were you using?

- Have you experienced any contraceptive failures (e.g., broken condom, missed birth control pills)?

**4. Medical History:**

- Can you provide more details about the acne capsules you are taking (e.g., the specific medication, dosage, and how long you have been using it)?

- Do you have any known allergies or previous medical conditions?

**5. Lifestyle Factors:**

- Do you smoke, consume alcohol, or use recreational drugs?

- What is your diet like, and do you engage in regular physical activity?

**6. Support System:**

- Do you have a support system in place (friends, family) for discussing this situation?

- Are you feeling anxious or stressed about the possibility of being pregnant?

**7. Future Considerations:**

- What are your thoughts on pregnancy? Are you considering continuing the pregnancy, or are you leaning towards termination if you are pregnant?

- Are you aware of the resources available for prenatal care or reproductive health services in your area?

Gathering this information would help in assessing her situation accurately and providing appropriate advice regarding next steps, including confirming pregnancy, considering options, and ensuring her health and well-being.”

# Appendix A7: ChatGPT-4o Response to Patient’s Revised Prompt relating to isotretinoin treatment in the context of possible pregnancy.

**ChatGPT Response**

*“*If you suspect that you might be pregnant and are taking isotretinoin for acne, it's important to take immediate steps to ensure your health and the health of your potential baby. Isotretinoin is known to be highly teratogenic, meaning it can cause serious birth defects. Here's a comprehensive guide on what to do:

1. Confirm the Pregnancy

- Home Pregnancy Test: Use a home pregnancy test to check for pregnancy. Follow the instructions carefully and use the test after a missed period for the most accurate result.

- Consult a Healthcare Provider: Regardless of the home test result, schedule an appointment with your healthcare provider to confirm the pregnancy with a blood test and clinical examination.

2. Discontinue Isotretinoin

- Stop the Medication: If you confirm that you are pregnant, you should immediately stop taking isotretinoin. This medication is known to cause severe birth defects and is contraindicated during pregnancy.

- Inform Your Healthcare Provider: Let your healthcare provider know about your isotretinoin use and pregnancy. They will provide guidance on how to safely discontinue the medication and discuss alternative treatments for acne if necessary.

3. Seek Prenatal Care

- Early Prenatal Appointment: Schedule an appointment with a healthcare provider for early prenatal care. This will allow for monitoring of your health and the development of the baby.

- Discuss Health History: Provide your healthcare provider with a full history of your isotretinoin use, including the duration and dosage.

4. Assess Acne Treatment Alternatives

- Safer Options: Discuss alternative acne treatments with your healthcare provider. There are safer options for managing acne during pregnancy, such as topical treatments that are not known to harm the developing fetus.

- Avoid Certain Treatments: Some acne treatments are not recommended during pregnancy, so it's important to choose options that are safe.

5. Adopt a Healthy Lifestyle

- Nutrition: Focus on a balanced diet and consider taking prenatal vitamins, particularly those with folic acid, to support a healthy pregnancy.

- Avoid Harmful Substances: Refrain from alcohol, tobacco, and illicit drugs. Limit caffeine intake and consult your healthcare provider before taking any over-the-counter medications or supplements.

6. Seek Support

- Educational Resources: Look for resources and support groups for expecting mothers. These can provide valuable information and support during your pregnancy.

- Mental Health: If you have concerns about your mental health or the impact of pregnancy on your well-being, discuss these with your healthcare provider.

Sources for More Information

- American College of Obstetricians and Gynecologists (ACOG): Provides information on pregnancy care and what to expect.

- Mayo Clinic: Offers resources on early pregnancy care and managing health conditions during pregnancy.

- Centers for Disease Control and Prevention (CDC): Provides guidelines on prenatal health and pregnancy safety.

Conclusion

If you think you might be pregnant and are taking isotretinoin, it is crucial to stop the medication immediately and consult with your healthcare provider. They will guide you through the process of managing acne safely during pregnancy and ensure you receive appropriate prenatal care to support a healthy pregnancy.*”*

# Appendix A8: ChatGPT-4o performs a consistency check on its prior response relating to isotretinoin use in the context of possible pregnancy.

“The statement provided is generally consistent and aligns well with established medical guidelines regarding isotretinoin and pregnancy. However, there are a few points that could be clarified or elaborated on for completeness:

1. **Timing of Pregnancy Testing**: The statement advises using a home pregnancy test after a missed period for the most accurate result. It could mention that some sensitive tests can detect pregnancy before a missed period, which may prompt earlier action.

2. **Alternative Treatments for Acne**: While it mentions that there are safer options for managing acne during pregnancy, it could be beneficial to specify that some commonly used topical treatments (like certain antibiotics or azelaic acid) are generally considered safer, while others (like salicylic acid in high concentrations) may not be recommended.

3. **Mental Health Support**: The mention of mental health is important, but it could emphasize that pregnancy can significantly affect mental health, and those with a history of depression or anxiety should particularly seek support early.

4. **Follow-Up Monitoring**: The statement suggests scheduling an early prenatal appointment but could highlight the importance of follow-up care for both maternal and fetal health, especially given the history of isotretinoin use.

Overall, while these points are not contradictions, they would enhance the clarity and thoroughness of the guidance provided.”

# Appendix A9: OpenBioLLM-70b response to initial prompt relating to isotretinoin use in the context of possible pregnancy.

“If you think you might be pregnant, it's important to take a few steps to ensure the health of you and your potential baby. Here are some recommendations: 1. Take a pregnancy test: You can purchase an over-the-counter pregnancy test at most drugstores. Follow the instructions on the packaging to determine if you are pregnant. 2. Schedule a prenatal appointment: If the pregnancy test is positive, make an appointment with your healthcare provider for a prenatal check-up. They will assess”

# Appendix A10: OpenBioLLM-70b response to a Redrafting Prompt to elicit additional information from a patient considering isotretinoin treatment in the context of possible pregnancy

“To make a good decision, I would need to know the following additional information: 1. Have you missed your period? 2. Are you experiencing any other symptoms of pregnancy such as breast tenderness, fatigue, or nausea? 3. When was your last menstrual period? 4. Have you taken a home pregnancy test? If yes, what were the results? 5. Are you currently using any form of contraception? 6. Have you had any previous pregnancies or abortions?”

# Appendix A11: OpenBioLLM-70b response to patient’s Revised Prompt relating to isotretinoin treatment in the context of possible pregnancy.

“If you think you might be pregnant, it's important to take a few steps to confirm and seek appropriate care. Here's what you should do: 1. Take a home pregnancy test: You can purchase a home pregnancy test from a pharmacy or drugstore. Follow the instructions on the package to determine if you are pregnant. 2. Schedule a prenatal appointment: If the home pregnancy test is positive, or if you're still unsure after taking the test, schedule an appointment with your”

# Appendix A12: OpenBioLLM-70b performs a consistency check on ChatGPT’s response to a patient considering isotretinoin treatment in the context of possible pregnancy.

“There are no inconsistencies or contradictions in the provided statement. The information is accurate and comprehensive, guiding individuals who suspect they might be pregnant while taking isotretinoin for acne. It emphasizes the importance of confirming pregnancy, discontinuing isotretinoin, seeking prenatal care, discussing alternative acne treatments, adopting a healthy lifestyle, and seeking support. The sources mentioned, such as ACOG, Mayo Clinic, and CDC, are reputable and provide reliable information on pregnancy care and safety.”

**(Supplemental File)**

# Table A13 Author Discipline, Institution, Location and Contribution.

| **Name** | **Discipline, content expertise** | **Institution** | **Geographical location** | **A description of the member’s contribution.** |
| --- | --- | --- | --- | --- |
| Padhraig Ryan | Researcher, pharmacist, machine learning engineer | Pharmaceutical Society of Ireland | Dublin, Ireland | Jointly initiated and authored each draft |
| Orla Davoren | Patient author | Patient author | Limerick, Ireland | Co-developed the clinical scenario and the prompts, interpreting responses from the LLM, reviewing drafts |
| Glyn Elwyn | Researcher, former primary care clinician. | The Dartmouth Institute for Health Policy & Clinical Practice | Vermont, USA | Jointly initiated and authored each draft |
