# Supplementary material for: Fact-Checking Large Language Model Responses to a Health Care Prompt: Comparative Study
Source: JMIR Form Res. 2026 Apr 15;10:e68223. doi: 10.2196/68223 (PMC13082570; doi:10.2196/68223)
Supplement: Multimedia Appendix 2 [file formative-v10-e68223-s002.doc]

**Table S1: Methodology checklist for GenAI study (based on Sallam et al. [14])**

| **Item** | **Issues considered in each item** | **Results** |
| --- | --- | --- |
| #1 Model | What is the model of the generative AI tool used for generating content, and what are the exact settings for each tool? | ChatGPT-4o and OpenBioLLM-70B  We used an Nvidia A100 Graphical Processing Unit to run the OpenBioLLM-70b model on Google Colab. This was accelerated using the 'unsloth' package 2025.3.18. |
| #2 Evaluation | What is the exact approach used to evaluate the content generated by the generative AI-based model and is it an objective or subjective evaluation? | Responses were evaluated by three pharmacists independently. This comprised fact-checking of healthcare statements. |
| #3a Timing | When is the generative AI model tested exactly and what are the duration and timing of testing? | ChatGPT-4o was used on 16th - 18th September 2024.  OpenBioLLM-70B was used on 6 January 2025. |
| #3b Transparency | How transparent are the data sources used to generate queries for the generative AI-based model? | Prompts were generated collaboratively with a patient author.  Prompts are evaluated based on the subject matter expertise of pharmacists, and literature search, where needed |
| #4a Range | What is the range of topics tested and are they intersubject or intrasubject with variability in different subjects? | 13 fact-checks pertain to a hypothetical patient use case involving isotretinoin.  Another 20 fact checks relate to disparate healthcare topics (see Appendix A2). |
| #4b Randomisation | Was the process of selecting the topics to be tested on the generative AI-based model randomized? | Non-randomised |
| #5 Individual | Is there any individual subjective involvement in generative AI content evaluation? If so, did the authors describe the details in full? | Objective assessment based on clinical knowledge. Details are provided in the results section. |
| #6 Count | What is the count of queries executed (sample size)? | 37 prompts were administered to each model.  13 prompts involve fact-checking the isotretinoin patient scenario.  20 prompts involve fact-checking a wider range of topics to assess generalisability.  Three prompts comprise the hypothetical patient's queries for the isotretinoin use case.  One prompt is a consistency check. |
| #7 Specificity of the  prompt or language | How specific are the exact prompts used? Were those exact prompts provided fully? Did the authors consider the feedback and learning loops? How specific are the language and cultural issues considered in the generative AI model? | The full text of all prompts is provided in the manuscript and appendices. |
